# Colorectal cancer-derived extracellular vesicles induce liver premetastatic immunosuppressive niche formation to promote tumor early liver metastasis

**DOI:** 10.1038/s41392-023-01384-w

**Published:** 2023-03-06

**Authors:** Xuyang Yang, Yaguang Zhang, Yang Zhang, Huifang Li, Li Li, Yangping Wu, Xiangzheng Chen, Lei Qiu, Junhong Han, Ziqiang Wang

**Affiliations:** 1 Colorectal Cancer Center, Department of General Surgery, State Key Laboratory of Biotherapy and Cancer Center, and Frontiers Science Center for Disease-related Molecular Network, West China Hospital, Sichuan University, Chengdu, 610041 Sichuan Province China; 2 State Key Laboratory of Biotherapy and Cancer Center, and Frontiers Science Center for Disease-related Molecular Network, West China Hospital, Sichuan University, Chengdu, 610041 Sichuan Province China; 3 Core Facility, Institute of Clinical Pathology, Department of Respiratory and Critical Care Medicine, and Department of Liver Surgery & Liver Transplantation, State Key Laboratory of Biotherapy and Cancer Center, West China Hospital, Sichuan University and Collaborative Innovation Center of Biotherapy, Chengdu, 610041 China

**Keywords:** Gastrointestinal cancer, Gastrointestinal cancer

**Dear Editor,**

To date, the effect of colorectal cancer (CRC)-derived extracellular vesicles (EVs) on liver pre-metastatic niche (PMN) remain incompletely understood.^1^ To investigate the role of CRC-derived EVs in the remodeling of the liver PMN, we isolated EVs from CT26 cell culture supernatant. The characteristics of EVs (the morphology, size, and markers) were identified by transmission electron microscopy (TEM), Nanoparticle Tracking Analysis (NTA), and western blotting (Supplementary Fig. S1a–e). Then, BALB/c mice with an intact liver immune status were pretreated with CRC-derived EVs and phosphate-buffered saline (PBS) for one month, and a liver metastasis model was established via spleen injection of tumor cells to determine whether EVs influence liver metastasis (Fig. 1a). Two hours after the operation, in vivo imaging indicated that the animal model was successfully established, and the tumor fluorescence intensity in the liver was consistent between the two groups (Supplementary Fig. S2a, b). Amazingly, the liver tumor fluorescence intensity in the EVs group was significantly stronger than that in the PBS group after 24 h (Fig. 1b; Supplementary Fig. S2c, d). The number of liver tumor nodules in the EVs group was also significantly higher than that in the PBS group on day 4 (Fig. 1c; Supplementary Fig. S2e, f). The efficacy of EVs in promoting tumor liver metastasis was consistent regardless of whether the spleen was being preserved when the animal model was established (Supplementary Fig. S3a–e).

**Fig. 1:**
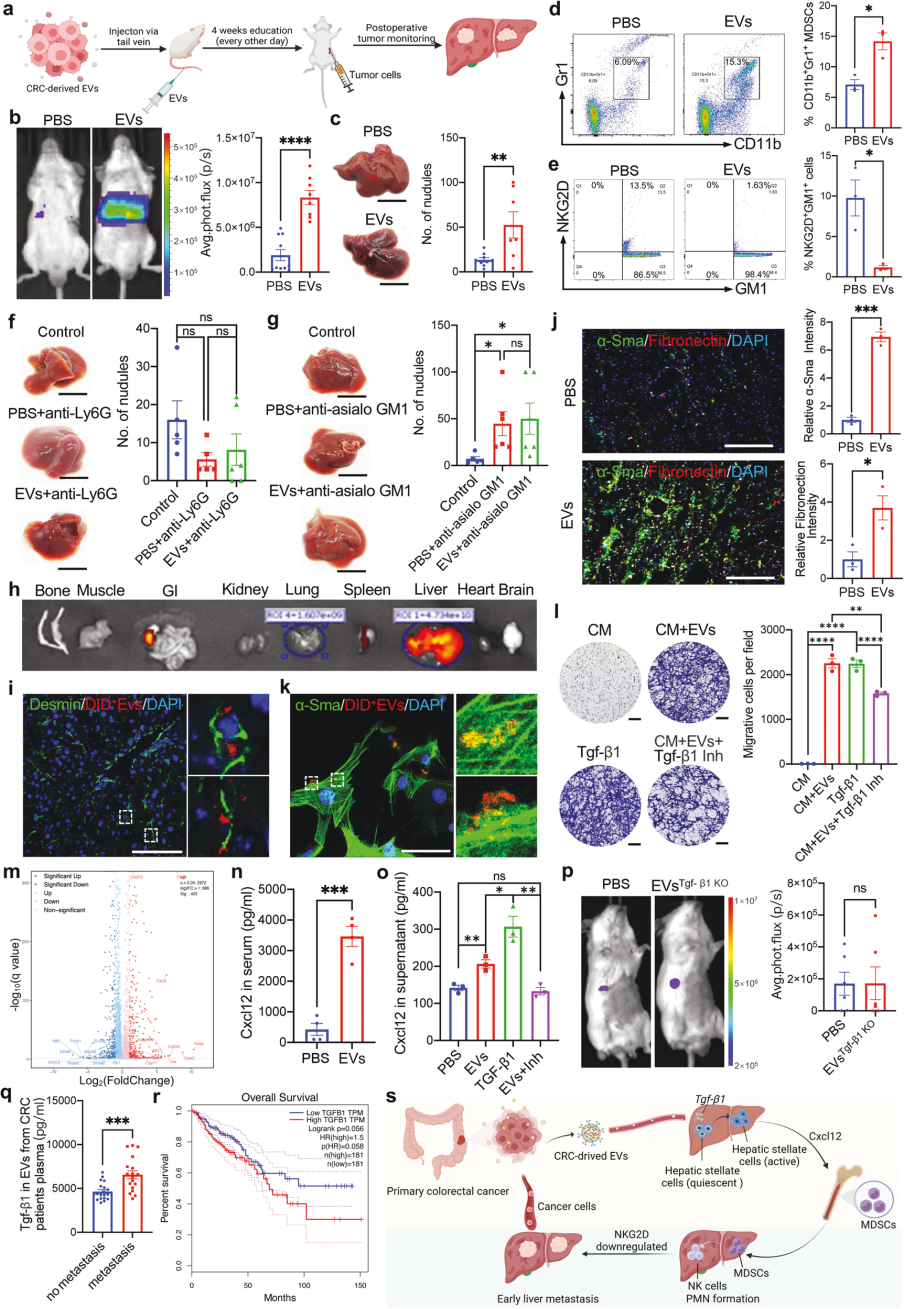
CRC-derived EVs initiate a sequential event to remodel the liver premetastatic niche and promote tumor liver metastasis. **a** Schematic study overview. **b** Left: Representative bioluminescence imaging showed that, compared with the PBS group (*n* = 10), the liver tumor fluorescence intensity was significantly stronger in the EVs group (*n* = 8) at 24 h after operation. Right: Quantifying tumor fluorescence intensity further confirmed that the tumor burden was heavier in the EVs group. The detail of the animal experiment was also shown in Fig. S2. **c** Left: Representative liver images showed more metastatic nodules on the liver surface in the EVs group. Right: Quantifying liver tumor nodules confirmed that the mean number of liver tumor nodules was significantly higher in the EVs group (13.33 vs. 52.43, *p* = 0.009). **d** Left: Representative image demonstrated that the CD11b^+^Gr1^+^ cells population screened by flow cytometry in the liver PMN after CRC-derived EVs education increased (*n* = 3 in the PBS group, *n* = 3 in the EVs group). Right: Quantitative analysis confirmed that the mean proportion of the CD11b^+^Gr1^+^ cells subset in the CD45^+^ lymphocyte population increased significantly in the EVs group (7.09% vs. 14.17%, *p* = 0.012). In rescue assay, after CRC-derived exsomal TGF-β1 knockout and HSCs-derived CXCL12 depletion, the mean proportion of CD11b^+^ Gr1^+^ cells subset was 8.86% in the EVs with CXCL12 depletion group and 7.92% in the EVs ^TGF-β1 KO^ group, respectively. Compared with the PBS group, there was no significant difference among the three group in Fig S4 **h**, **i**. **e** Left: Representative image demonstrated that, in the liver PMN, the expression of NKG2D in NK cells was downregulated after CRC-derived EVs education. Right: Quantitative analysis confirmed that compared with the PBS group (*n* = 3), NKG2D expression level in NK cells was significantly downregulated in the EVs group (*n* = 3) (8.5% vs. 1.15%, *p* < 0.05). **f** Left: Representative liver images showed the metastatic nodules on the liver surface in the control group (*n* = 5), PBS group (*n* = 5), and EVs group (*n* = 6). Right: The mean number of liver tumor nodules was 16 in the control group, 5.6 in the PBS group, and 8.2 in the EVs group. Statistical analysis showed that, for tumor burden, there were no significant differences between the PBS group and the EVs group after the CD11b^+^Gr1^+^ cells subset depletion (5.6 vs. 8.2, *p* = 0.608). The tumor nodule number in the PBS group and the EVs group tended to decrease compared with that in the control group. **g** Left: Representative liver images showed the metastatic nodules on the liver surface in the control group (*n* = 5), PBS group (*n* = 6), and EVs group (*n* = 6). Right: The mean number of liver tumor nodules was 7 in the control group, 44.67 in the PBS group, and 50 in the EVs group. Statistical analysis showed that, for tumor burden, there were no significant differences between the PBS group and the EVs group after CD45^+^GM1^+^ cells subset depletion (44.67 vs. 50, *p* = 0.805). Tumor nodule number was significantly higher in the PBS group and the EVs group than in the control group. These results demonstrated that the regulated NK cells in the liver PMN remodeled by EVs have an important role in promoting tumor liver metastasis. **h** Representative imaging showed that DID dye–labeled CRC-derived EVs mainly accumulated in the liver at 2 and 24 h after injection. **i** Immunofluorescence confocal analysis demonstrated that DID^+^ EVs were mainly absorbed by Desmin^+^ HSCs. **j** Left: Immunofluorescence confocal analysis demonstrated that HSCs were massively activated and significantly increased their expression of α-SMA and fibronectin after CRC-derived EVs education for 4 weeks. Right: Statistical analysis further confirmed that α-SMA and fibronectin intensity was significantly increased after CRC-derived EVs education for 4 weeks. **k** Representative imaging showed that quiescent HSCs absorbed EVs in vitro. **l** Left: A co-culture assay showed that tumor migration was enhanced after HSCs were co-cultured with exogenous Tgf-β1 and CRC-derived EVs and transformed into CAFs. Right: Statistical analysis further confirmed that after co-culture with exogenous Tgf-β1 and CRC-derived EVs, CAFs enhanced tumor migration, and the effect was abolished by Tgf-β1 inhibitor. **m** The volcano map showed that chemokines such as CXCL 12 were highly expressed. **n** ELISA showed that CXCL12 was highly expressed in serum after mice were educated with CRC-derived EVs for 4 weeks. **o** ELISA confirmed that EVs and exogenous Tgf-β1 enhanced the expression of CXCL12 in the cell supernatant of CAFs, and the expression of CXCL12 was inhibited by Tgf-β1 inhibitor. **p** Left: After the mice were educated with Tgf-β1–knockout EVs for 4 weeks, a CRC liver metastasis model was established to confirm the effect of Tgf-β1 knockout. Representative liver images showed liver tumor fluorescence intensity in the PBS group (*n* = 6) and the EVs Tgf-β1 group (*n* = 6). Right, There was no significant difference in tumor burden between the PBS group (*n* = 6) and EVs Tgf-β1 group (*n* = 6) at 24 h after operation. The detail of the animal experiment was also shown in Fig. S13. **q** The expression level of exosomal TGFB1 in plasma was significantly higher in CRC patients with synchronous liver metastasis (*n* = 18) than in those without metastasis (*n* = 20). **r** Survival analysis suggested that patients with higher TGFB1 expression levels had worse overall survival. **s** The working model in this study (https://biorender.com/). **c**, **f**, **g** scale bar: 1 cm; **I**, **j**, **l**, scale bar: 100 μm; **k** scale bar: 50 μm; GI gastrointestinal tract, Inh inhibitor, CM culture medium, HSCs hepatic stellate cells. Data are shown as means ± SD. ‘ns’ means no significance, *p < 0.05, **p < 0.01, ***p < 0.001, ****p < 0.0001

Since we confirmed that CRC-derived EVs contributed to tumor early liver metastasis, we speculated that EVs induced liver PMN transformation into the immunosuppressive niche. Increasing evidence confirmed that myeloid-derived suppressor cells (MDSCs) are major regulators of the immune response in pathological conditions and key contributors to tumor progression.^2^ We analyzed the evolution of MDSCs by flow cytometry in the liver PMN before and after EVs education. We found that EVs education significantly increased the CD11b^+^Gr1^+^ cells (especially the CD11b^+^Ly6G^high^Ly6C^low^ subset) number in the liver PMN (Fig. 1d; Supplementary Fig. S4a–e).

Traditionally, MDSCs facilitates tumor immune escape by inhibiting T-cell function.^2^ However, the mechanism by which the recruited MDSCs induce the liver PMN to enter an immunosuppressive state remains to be elucidated. As a prominent cellular component of the innate immune system, natural killer (NK) cells play a vital role in the early monitoring of tumor immune escape and the regulation of distant metastases.^3^ Our xenograft assay demonstrated that tumor cells evaded the immune system’s surveillance, suggesting that NK cells had decreased cytotoxicity after EVs education. Moreover, we further confirmed that the expression of NKG2D, the main functionally activated receptor in NK cells, was significantly reduced after EVs education, although the number of NK cells did not change (Fig. 1e; Supplementary Fig. S4f, g). Collectively, our findings suggested that CRC-derived EVs induced liver immunosuppressive PMN formation via recruiting MDSCs and impair NK-cell cytotoxicity.

To further evaluate whether the recruited MDSCs are the key cellular components during liver PMN formation and whether NK cells play a vital role in immune surveillance, anti-Ly6G and anti-asialo GM1 antibodies were injected intraperitoneally to ablate the recruited MDSCs and residential NKs in the liver PMN after EVs education. After MDSCs and NK cells had been successfully ablated (Supplementary Fig. S5b–e; Supplementary Fig. S6b–e), the liver metastatic model was established to confirm the effect of MDSCs and NK cells depletion (Supplementary Fig. S5a; Supplementary Fig. S6a). Interestingly, after MDSCs depletion, no significant difference was found in tumor burden between the two groups. Furthermore, the number of tumor nodules in both groups tended to decrease compared with that in the control group (Fig. 1f), suggesting that MDSCs depletion can reverse the effect of EVs on promoting tumor liver metastasis. On the other hand, after NK cells depletion, no significant difference in tumor burden was found between the two groups, but the number of tumor nodules in the two groups was significantly higher compared with that in the control group (Fig. 1g), indicating that NK cells ablation can enhance the effect of EVs on promoting tumor liver metastasis. Taken together, these results indicated that recruited MDSCs had an important role in the liver immunosuppressive PMN formation and that the function of NK cells was inhibited by MDSCs, resulting in the failure of immune surveillance.

To further explore how EVs initiate the sequential events of liver PMN formation, we investigated the distribution of EVs in vivo. We found that EVs mainly accumulated in the liver and were taken up by hepatic stellate cells (HSCs) (Supplementary Fig. S7a–d; Fig. 1h, i). It is well-known that the activation of HSCs transdifferentiation of quiescent cells into proliferative, fibrogenic myofibroblasts is a central driver of liver extracellular matrix remodeling.^4^ As expected, after EVs education for one month, HSCs were massively activated and significantly increased α-SMA and fibronectin secretion in vivo (Supplementary Fig. S7e; Fig. 1j). After co-culture with EVs in vitro, the markers of tumor-associated fibroblasts (CAFs), including α-SMA, vimentin, and FAP, were significantly increased in activated HSCs (Supplementary Fig. S7f; Supplementary Fig. S8a–d; Fig. 1k). These results revealed that EVs were mainly taken up by HSCs to trigger HSCs transformation into CAFs, which secrete fibronectin and remodel the liver PMN.

Our previous study revealed that gastric cancer-derived EVs carrying Tgf-β1 remodel the preperitoneal PMN and promote tumor peritoneal metastasis.^5^ In this study, we also confirmed that Tgf-β1 was the major component in EVs that induced HSCs transformation into CAFs phenotype (Supplementary Fig. S8c, d). In vitro co-culture showed that CAFs further enhanced tumor migration, and the effect was abolished by the Tgf-β1 inhibitor (Fig. 1l). Furthermore, RNA-sequencing analysis and ELISA confirmed that, as an essential signaling pathway for liver PMN remodeling, the chemokine CXCL12/CXCR7 axis was activated after HSCs co-incubated with EVs (Fig. 1m–o; Supplementary Fig. S9a–d; Supplementary Fig. S10a–d; Supplementary Fig. S11 a-g; Supplementary Fig. S12a–h). To confirm the direct link between HSCs-derived CXCL12 and MDSCs, HSCs-derived CXCL12 was depleted to confirm the proportion of recruited CD11b^+^ Gr1^+^ cells subset after EVs education. There was no significant difference between the PBS group and EVs with CXCL12 depletion group (Supplementary Fig. S4h, i). Thus, we propose that HSCs are transformed into CAFs in response to EVs, leading to increased CXCL12 secretion, which in turn recruits MDSCs for remodeling of the liver immunosuppressive PMN.

Next, we used the CRISPR–Cas9 method to knock out CRC-derived exosomal Tgf-β1 and further confirmed the Tgf-β1 enrichment in EVs is a key cytokine that induces liver PMN formation (Supplementary Fig. S1f–g; Fig. 1p; Supplementary Fig. S13a–f). Moreover, the expression of TGF-β1 from circulating EVs was significantly higher in CRC patients with synchronous liver metastasis than in those without liver metastasis, and patients with higher TGFB1 expression had worse survival (Fig. 1q, r; Supplementary Fig. S13g–i). Collectively, these results demonstrated that Tgf-β1 enriched in EVs played a vital role in inducing liver PMN formation and promoting tumor liver metastasis, and CRC-derived exosomal TGF-β1 has potential as a molecular marker.

In conclusion, CRC-derived EVs carrying Tgf-β1 activate the HSCs chemokines signaling pathway and induce HSCs to transform into CAFs phenotype. After HSCs activation, MDSCs are further recruited into the liver PMN to inhibit NK-cell cytotoxicity by downregulating the expression of NKG2D. Finally, CRC-derived EVs remodel the liver PMN and promote tumor liver metastasis (Fig. 1s).

## Supplementary information

Supplementary marterials

## Data Availability

All relevant data are placed within the article and Supplementary Files, or available from the corresponding author upon reasonable request.
