# Supplementary material for: Colorectal cancer-derived extracellular vesicles induce liver premetastatic immunosuppressive niche formation to promote tumor early liver metastasis
Source: Signal Transduct Target Ther. 2023 Mar 6;8:102. doi: 10.1038/s41392-023-01384-w (PMC9988828; doi:10.1038/s41392-023-01384-w)
Supplement: Supplementary file 1 — Supplementary marterials [file 41392_2023_1384_MOESM1_ESM.docx]

**Supplementary Materials for**

**Colorectal cancer–derived extracellular vesicles induce liver premetastatic immunosuppressive niche formation to promote tumor early liver metastasis**

Xuyang Yang**^1#^**, Yaguang Zhang**^2#^**, Yang Zhang^1^, Huifang Li^3^, Li Li^4^, Yangping Wu^5^, Xiangzheng Chen^6,7^, Lei Qiu^2^, Junhong Han**^2^***, Ziqiang Wang**^1^***

Correspondence to: Ziqiang Wang, E-mail: [wangziqiang@scu.edu.cn](mailto:wangziqiang@scu.edu.cn); Junhong Han, E-mail: [hjunhong@scu.edu.cn](mailto:hjunhong@scu.edu.cn)

This file includes:

Materials and Methods

Abbreviations

Supplementary S1 to S13

Supplementary Table 1

References

**Materials and Methods**

**Cell lines and cell culture**

To investigate the effect of CRC-derived EVs on the liver immune microenvironment, mice with an intact liver immune status are needed. Thus, BALB/c mice without a defective immune system were used to establish an animal model in our study. Correspondingly, a mouse colon cancer cell line (CT26. WT), which was purchased from the American Type Culture Collection (ATCC), was used to perform cell experiments ex vivo*.* CT26. WT cells were cultured in RPMI 1640 medium (Gibco, USA) supplemented with 10% EVs-depleted fetal bovine serum (System Biosciences, CA, USA) and 1% penicillin–streptomycin solution (Gibco, USA). Primary cultured mouse hepatic stellate cells (HSCs) were obtained from Procell Life Science & Technology Co., Ltd. (Wuhan, China). HSCs were cultured in HSCs complete culture medium (Procell Life Science & Technology Co., Ltd., Wuhan, China). All cell lines were maintained in a humidified incubator at 37°C with 5% CO_2_. All cell lines were tested for mycoplasma contamination and short tandem repeat (STR) DNA profiling before use.

**Isolation of EVs from the medium**

To obtain CT26-derived EVs, CT26 cells were cultured in media supplemented with 10% EVs-depleted fetal bovine serum (System Biosciences, CA, USA). Cell supernatant was collected from 48-h cell culture and subjected to isolation of EVs. Both the differential ultracentrifugation method and ultrafiltration plus size exclusion chromatography (SEC) were carried out to collect EVs.

The differential ultracentrifugation method was conducted as follows. The initial cell supernatant fractions were collected and pelleted by centrifugation at 300 × *g* for 10 min. After removing the precipitate, the supernatant was centrifuged at 10000 × *g* for 30 min. The resulting supernatant was further centrifuged at 100,000 × *g* for 70 min. After that, EVs pellets were harvested and resuspended in 30 mL of phosphate-buffered saline (PBS, pH value 7.2–7.4) and collected by ultracentrifugation at 100,000 × *g* for 70 min (BECKMAN SW32ti rotor). The procedure of ultrafiltration plus size exclusion chromatography (SEC) was conducted as follows. Cell supernatant fractions were collected and pelleted by centrifugation at 2000 × *g* for 30 min. After filtering with a 0.22-µm filter, the supernatant was concentrated using a 100-kD ultrafiltration tube (Millipore, USA) by centrifugation at 2000 × *g* for 25 min. The concentrated supernatants were purified using Exosupur® columns (Echobiotech, Beijing, China) in accordance with the manufacturer's instructions. Briefly, a 1-mL sample was loaded into the Sepharose-based column. After all samples were added to the column, 1.5 mL PBS was added to the column to eluate EVs. The eluent was discarded. Then, a total of 2.5 mL PBS was added to the column. Each 500 μL of the effluent represented one fraction, and a total of five specific fractions enriched with EVs were collected. The isolated EVs were resuspended in PBS buffer. The quantities of EVs used for the in vivo and in vitro experiments were measured using a BCA protein assay kit (Beyotime, Shanghai, China). EVs were subpackaged and preserved at −80°C until use.

**Identification of EVs**

The size distribution and concentration of EVs were determined by Nanoparticle Tracking Analysis (NTA) (ZetaView PMX 110, Particle Metrix, Meerbusch, Germany) in accordance with the manufacturer’s instructions. CRC-derived EVs were resuspended in 1 mL PBS and mixed, then the diluted EVs were injected into the ZetaView PMX 110. Particles were tracked and the size of particles was measured based on Brownian motion and the diffusion coefficient. Filtered PBS was used as a control. Data were analyzed by the corresponding software ZetaView 8.04.02 SP2. Western blotting was performed to identify the positive and negative markers of EVs. The isolated EVs were suspended in RIPA lysis buffer and incubated on ice for 10 min. After measuring the total protein concentration by the BCA protein assay kit, the samples were subjected to SDS–PAGE gel electrophoresis and western blot analysis with specific antibodies. EVs proteins were detected using the following antibodies: CD9 (1:1000, Abcam, MA, USA), CD81 (1:1000, Abcam, MA, USA), TSG101 (1:1000, Abcam, MA, USA), GRP94 (1:1000, Affinity, China), Calnexin (1:1000, BOSTER, China), and EPCAM (1:1000, BOSTER, China).

Transmission electron microscopy (TEM) was performed to investigate the morphological characteristics of EVs. Briefly, EV samples were added to the sealing membrane, and copper mesh was used to cover the sample. Next, 2% phosphotungstic acid solution was added to negatively stain the samples for 3 min at room temperature. After drying at room temperature for 3 min, the dried samples were observed and photographed by TEM (JEM-1400FLASH, JEOL, Tokyo, Japan).

**EVs labeling and treatment**

To observe EVs distribution in vivo and cellular uptake in cell cultures, purified EVs were fluorescently labeled using DiD red fluorescent dye (Sigma, St. Louis, Missouri, USA). The method was performed in accordance with the manufacturer’s instructions. Briefly, 5 μL of DiD membrane dye was added to 1 mL of EVs suspension (1:200) and mixed well. Next, the mixed suspension was incubated at room temperature for 20 min. After that, DiD dye–labeled EVs were filtered with a 0.22-μm Millipore filter membrane and stored at −20°C until use.

To confirm the distribution of EVs in vivo, 100 µL DiD-labeled EVs were injected into male BALB/c mice (BEIJING HFK BIOSCIENCE CO., LTD., Beijing, China) via a tail vein. Twenty-four hours after the injection, in vivo imaging and immunofluorescence staining were used to evaluate EVs distribution in vivo and confirm EVs uptake by cells. DiD red fluorescent dye diluted with PBS was used as a negative control. According to the EVs tracking assay in vivo, we confirmed that CRC-derived EVs were mainly taken up by HSCs. An in vitro immunostaining assay was performed to further verify whether HSCs take up EVs. Then, primary HSCs were isolated from a mouse liver and plated in cell climbing slices at a density of 2 × 10^4^. HSCs were incubated with DiD-labeled EVs for 24 h at 37°C, washed twice with PBS, and fixed with 4% formaldehyde in PBS for 20 min at room temperature. Then, the cells were incubated with primary antibodies against a-SMA (1:200, Cell Signaling Technology, Inc., USA) and with Alexa Fluor 488–labeled secondary antibodies (1:200, USA). The cell nuclei were stained with DAPI (Dojindo, Japan). The cellular uptake of DiD-labeled EVs was analyzed using a confocal laser microscope (Zeiss, Oberkochen, Germany).

**Animal experiment**

Male BALB/c mice (6–8 weeks old) were purchased from Beijing HFK Bioscience Co., Ltd. (Beijing, China) and housed in a pathogen-free animal facility with access to clean food and water. All animal experimental procedures followed protocols approved by the Animal Care and Use Committees of West China Hospital, Sichuan University. To further evaluate the role of EVs in distant liver metastasis, BALB/c male mice were initially pre-educated with CRC-derived EVs via the tail vein every other day for four consecutive weeks (10 µg of EVs in 200 µL of sterile PBS per mouse). In the control group, mice were synchronously pre-educated with 200 µL of PBS via the tail vein. After 4 weeks of education, the mice were injected intrasplenically with 5 × 10^5^ luciferase-labeled CT26 cells to establish a metastatic liver model. To avoid the interference of fluorescence from tumor cells in the spleen, two-thirds of the spleen were resected 3 min after tumor cell injection. Then, liver metastasis was detected by in vivo luciferase-based noninvasive bioluminescence imaging using the IVIS Lumina II platform (PerkinElmer) and measured by hematoxylin and eosin (H&E) staining of liver paraffin-embedded sections, accounting for the number of nodules on the liver surface.

To further verify the role of MDSCs and natural killer (NK) cells in the liver PMN, a rescue assay was performed with antibody-mediated depletion of MDSCs and NK cells. After the mice had been pre-educated with CRC-derived EVs for four consecutive weeks, anti-Ly6G (clone 1A8, BioXCell, West Lebanon, NH) was injected intraperitoneally twice weekly (100 µg/mouse) for one week to deplete MDSCs in the liver PMN^1^. Similarly, after pre-education with CRC-derived EVs for four consecutive weeks, the mice were intraperitoneally administered 200 µL anti-Asilao GM1 (FUJIFILM Wako Chemicals U.S.A. Corp) (100 µL/mouse, twice a week) to abolish NK cells in the liver PMN^2^. Then, a CRC liver metastatic model was established to confirm the effect of MDSCs and NK cells depletion on CRC-derived EVs promotion of CRC liver metastasis.

**Flow cytometry analysis**

Flow cytometry was used to explore the effect of CRC-derived EVs on the liver tumor premetastatic immunosuppressive microenvironment. For liver tissue dissociation and single-cell isolation, fresh liver tissue was immediately collected when the mice were sacrificed. Then, the liver was cut into small pieces (1–3 mm^3^) and incubated in dissociation solution with collagenase type I (Roche), collagenase type IV (Roche), and DNase (Roche) (the solution concentrations were 2, 2, and 1 mg/mL, respectively). The cell suspension was incubated for 20–30 min at room temperature on a shaker and filtered through a 70-µm strainer. The cells were washed in PBS and incubated with anti-CD45-Super bright 702 (2:100, Biolegend), anti-CD11b-APC-Cy7 (1.25:100, Biolegend), anti-Gr1-Alexa Fluor® 700 (2:100, Biolegend), anti-Ly-6C-FITC (0.5:100, Biolegend), anti-Ly-6G-APC (0.3:100, Biolegend), anti-Asialo-GM1-PE (1.25:100, Biolegend), and anti-NKG2D-FITC (2:100, Biolegend) antibodies at predetermined saturating concentrations. At least 1,000,000 cells were collected to acquire data on a BD FACS Canto cytometer, and were further analyzed using FlowJo software (Tree Star Inc.). To confirm the direct link HSCs-derived CXCL12 and recruited MDSCs in liver PMN, mice were pretreated with EVs via tail vein and simultaneously pretreated with anti-mouse CXCL12 monoclonal antibody (0.5mg/ml, R&D Systems, Minneapolis, MN) via intraperitoneal injection. After pretreatment, the fresh liver tissue was collected to prepare single-cell suspension and analyzed by flow cytometry. Meanwhile, mice were pretreated with EVs without TGF-β1 to confirm the effect of exosomal TGF-β1 on MDSCs recruitment.

**RNA extraction and real-time quantitative PCR assay**

First, mice were sacrificed after CRC-derived EVs education for 4 weeks, and fresh liver samples were collected. Then, we used liquid nitrogen to grind fresh liver tissue with a mortar and pestle. Total RNA was extracted using TRIzol reagent (Invitrogen) based on the manufacturer's protocol. Briefly, a total of 50 mg tissue powder was collected into a nuclease-free EP tube, and 1 mL TRIzol reagent was added. Homogenized samples were incubated for 5 min, and 200 µL chloroform (200 µL/1 mL TRIzol reagent) was added. Then, the EP tube was violently shaken for 15–20 s and placed at room temperature for 3 min. After centrifuging at 12,000 × *g* for 15 min at 4°C, the aqueous phase was transferred into a new nuclease-free EP tube with 500 µL isopropanol (500 µL/1ml TRIzol) and incubated for 30 min. After that, the pellets were collected by centrifuging at 12,000 × *g* for 10 min at 4°C and washed twice with 75% ethanol. The resulting pellets were dissolved in RNase-free water for further assays. Reverse transcription was carried out using PrimeScript RT reagent Kit with gDNA Eraser (Takara) in accordance with the manufacturer’s protocol. Quantitative PCR using SYBR Green Supermix (Bio-Rad) was performed using CFX96 Real-Time PCR System (Bio-Rad). The relative expression levels of target genes were normalized to that of the internal control Actin. The primers used in this study are listed in Table S1.

**HSCs gene expression analysis**

To further investigate the mechanism involved in liver PMN formation by HSCs, mouse primary HSCs were educated with CRC-derived EVs in vitro and subjected to gene expression analysis by RNA sequencing. A total of 2 × 10^5^ HSCs were plated in 6-well microplates in 2 mL of HSCs special medium and incubated for 24 h. Then, 100 µg CRC-derived EVs were added. After 48 h of incubation, total RNA was directly extracted from cell layers using TRIzol™ Reagent (Invitrogen, CA, USA) in accordance with the manufacturer’s protocol. The assay was performed in triplicate. The quality of extracted RNA, including RNA purity, quantification, and integrity, was assessed using a NanoDrop 2000 spectrophotometer (Thermo Scientific, USA) and an Agilent 2100 Bioanalyzer (Agilent Technologies, Santa Clara, CA, USA). In accordance with the manufacturer’s instructions, the VAHTS Universal V6 RNA-seq Library Prep Kit was used to construct the libraries. The libraries were then sequenced on the Illumina NovaSeq 6000 platform. RNA sequencing analysis was conducted by OE Biotech Co., Ltd. (Shanghai, China).

***Tgf-β1* gene knockout**

Genes were knocked out using the CRISPR–Cas9 system. Cas9/sgTgfb1 vectors were transiently transfected into CT26 by Lipo3000 reagent. Positively transfected cells were selected with 8 μg/mL puromycin after 48 h of transfection. Monoclonal screening was performed after puromycin screening, and then monoclonal cells were identified by western blot and sequencing (sgTgfb1_1#: AGCACTAGAAGCCACGGGAGTGG; sgTgfb1_2#: AGCGGACTACTATGCTAAAGAGG).

**Clinical prognostic significance of TGFB1 in patients with CRC**

To extend our findings to human cancers, we investigated the expression of *TGFB1* in CRC and normal tissue, and its prognostic significance. Gene Expression Profiling Interactive Analysis 2 (GEPIA2) (http://gepia2.cancer-pku.cn/#index) is an online analytical tool for users to analyze RNA-sequencing expression data, and contains thousands of tumors and normal tissue sample data from The Cancer Genome Atlas (TCGA) and the Genotype-Tissue Expression (GTEx) websites^3^. In this study, we used GEPIA2 to analyze the mRNA levels of *TGFB1* in CRC tissue and normal tissue and performed Student’s *t* tests. |Log2FC|>1 and *p*<0.01 were considered significant. The mRNA levels of *TGFB1* among colorectal cancer tissues at different clinical stages were also analyzed. Furthermore, overall survival (OS) and disease-free survival (DFS) were analyzed using Kaplan–Meier curves. The median was selected as the group cutoff for the survival plot. The Human Protein Atlas (https://www.proteinatlas.org/) is an online database for human proteins, including immunohistochemistry profiles and transcriptome profiles^4^. In our study, the immunohistochemical images were downloaded to compare the expression of TGFb1 between normal colon tissues and colorectal cancer tissues. We also collected blood samples from patients with or without synchronous liver metastasis to investigate the expression level of exosomal TGFB1 and CXCL12 in plasma. All clinical tissues were obtained from the West China Hospital (Chengdu, China). All patients signed informed written consent with the approval of the Biological and Medical Ethics Committee of West China Hospital.

**Statistical analysis**

The statistical analyses were performed by SPSS software version 22.0 (IBM Inc., Armonk, NY, USA) and GraphPad Prism 8.0 software (GraphPad Prism Software, Inc., San Diego, CA, USA). Continuous variables are expressed as the mean and standard deviation. The unpaired Student’s *t* test was used to examine the statistical significance between the two groups, and one-way analysis of variance was used for the statistical analysis of more than two groups. A *p* value < 0.05 (*), *p* value < 0.01 (**), *p* value < 0.001 (***), or *p* value < 0.0001 (****) was considered statistically significant.

**Abbreviations used in this paper:** CRC, colorectal cancer; EVs, extracellular vesicles; PMN, premetastatic niche; TEM, transmission electron microscopy; NTA, Nanoparticle Tracking Analysis; H&E, hematoxylin and eosin stain; PBS, phosphate-buffered saline; HSCs, hepatic stellate cells; MDSCs, myeloid-derived suppressor cells; NK, natural killer; Tgf-β1, transforming growth factor-β1; CAFS, cancer-associated fibroblasts

**Supplemental Figures**

**Supplementary S1 ：**

**
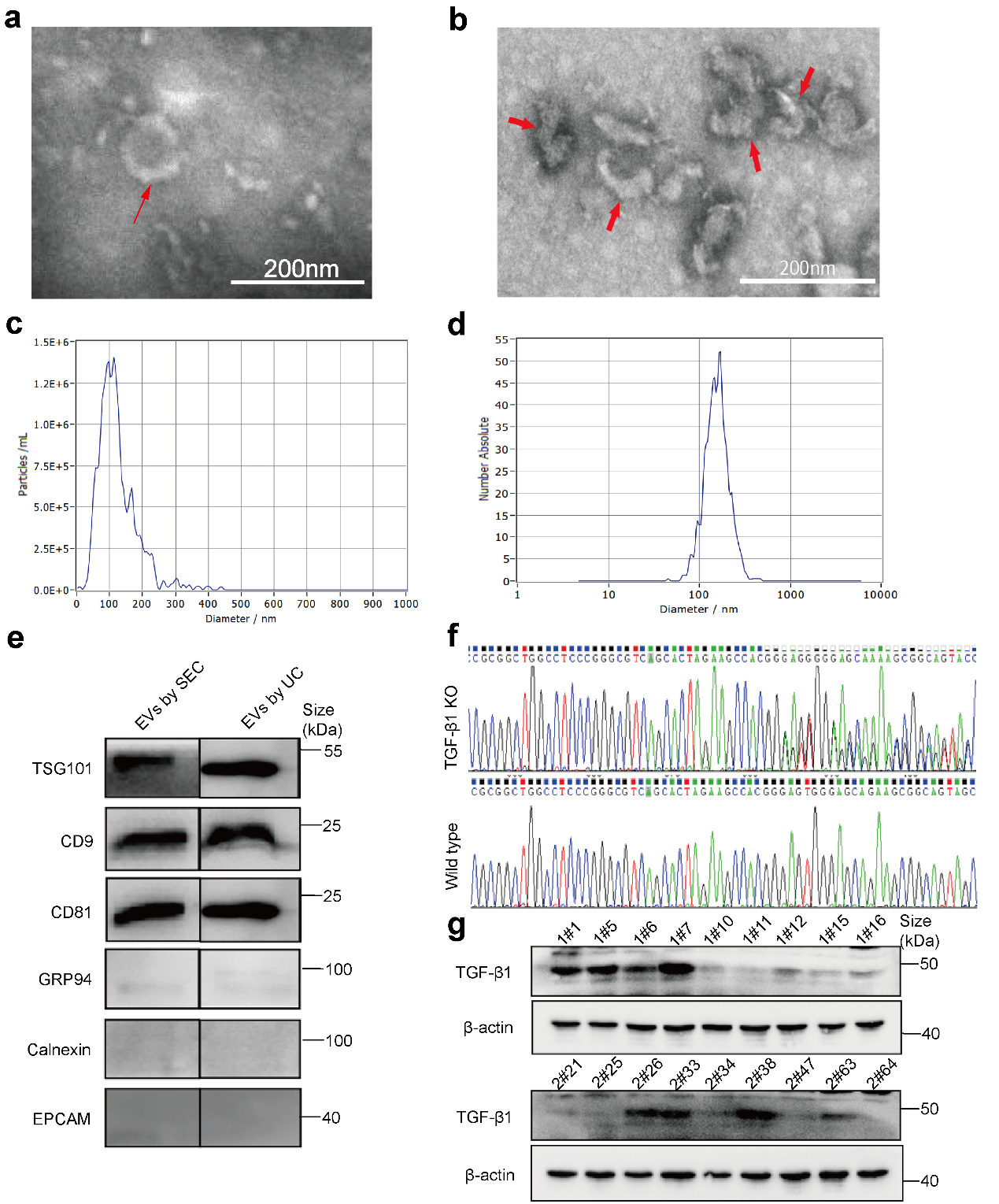
**

**Fig. S1: Characterization of CT26-derived EVs.** a/b, Transmission electron microscopy (TEM) showed that CT26-derived EVs had a typical “saucer-like” double-layer membrane structure (a, EVs isolated by SEC; b, EVs isolated by UC); c/d, The Nanoparticle Tracking Analysis (NTA) showed that the diameter of CT26-derived EVs ranged from 30 to 150 nm (a, EVs isolated by SEC; b, EVs isolated by UC). The median diameter of EVs was 110 nm when isolated by SEC and 130 nm when isolated by UC. e, Western blotting showed that CT26-derived EVs expressed positive protein markers, including TSG101, CD9, and CD81, while negative protein markers, including GRP94, Calnexin, and EPCAM, were not detected. f, Sanger sequencing verified the successful editing of Tgf-β1 target genes; g, The expression of Tgf-β1 in monoclonal cells was verified by western blotting.

**Supplementary S2 ：**
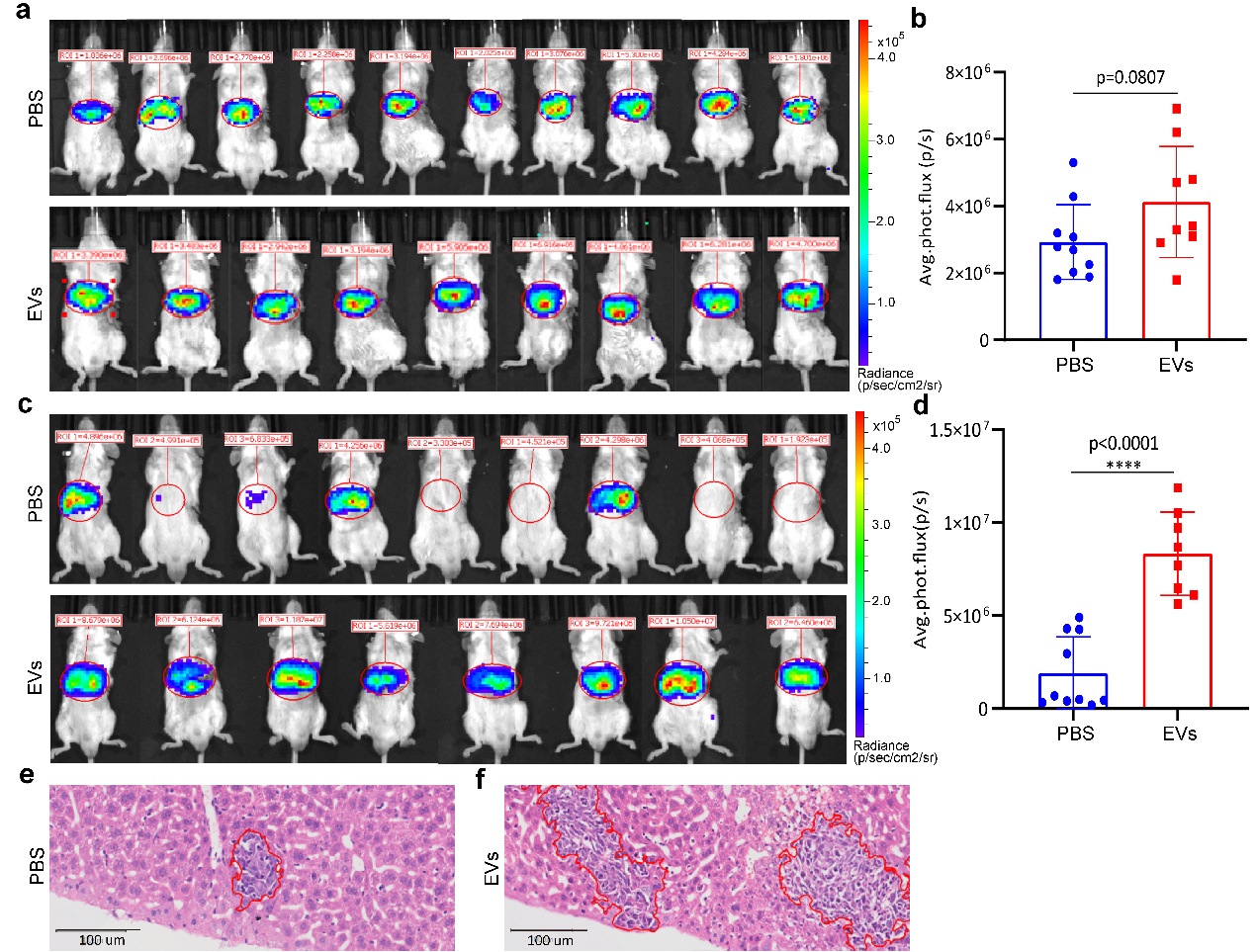


**Fig. S2: CRC-derived EVs promote tumor cells early liver metastasis.** After mice were educated with CRC-derived EVs for 4 weeks, a CRC liver metastasis model was established without spleen preservation. a, In vivo bioluminescence imaging confirmed that the CRC liver metastasis model had been successfully established in both the PBS group (n = 10) and the EVs group (n = 9). b, Quantifying tumor fluorescence intensity showed no significant difference in tumor burden between the two groups at 2 h after the operation, which indicated that the initial liver tumor burden was consistent between the two groups. c, In vivo bioluminescence imaging showed that, compared with the PBS group (n = 10), the liver tumor fluorescence intensity was significantly stronger in the EVs group (n = 8) at 24 h. d, Quantifying tumor fluorescence intensity further confirmed that the tumor burden was heavier in the EVs group. This result demonstrated that tumor cells in the EVs group continued to colonize and grow in the liver, while tumor cells in the PBS group had been killed in a short time. e/f, The mice were sacrificed on day 4 after the operation. The liver tissue was fixed in formalin and embedded in paraffin. Liver tissue were stained with H&E. H&E staining results confirmed that the white nodules on the liver surface were tumor nodules in the two groups. Data are shown as means ± standard deviations. **p* < 0.05; ** *p* < 0.01, *** *p* < 0.001.

**Supplementary S3 ：**


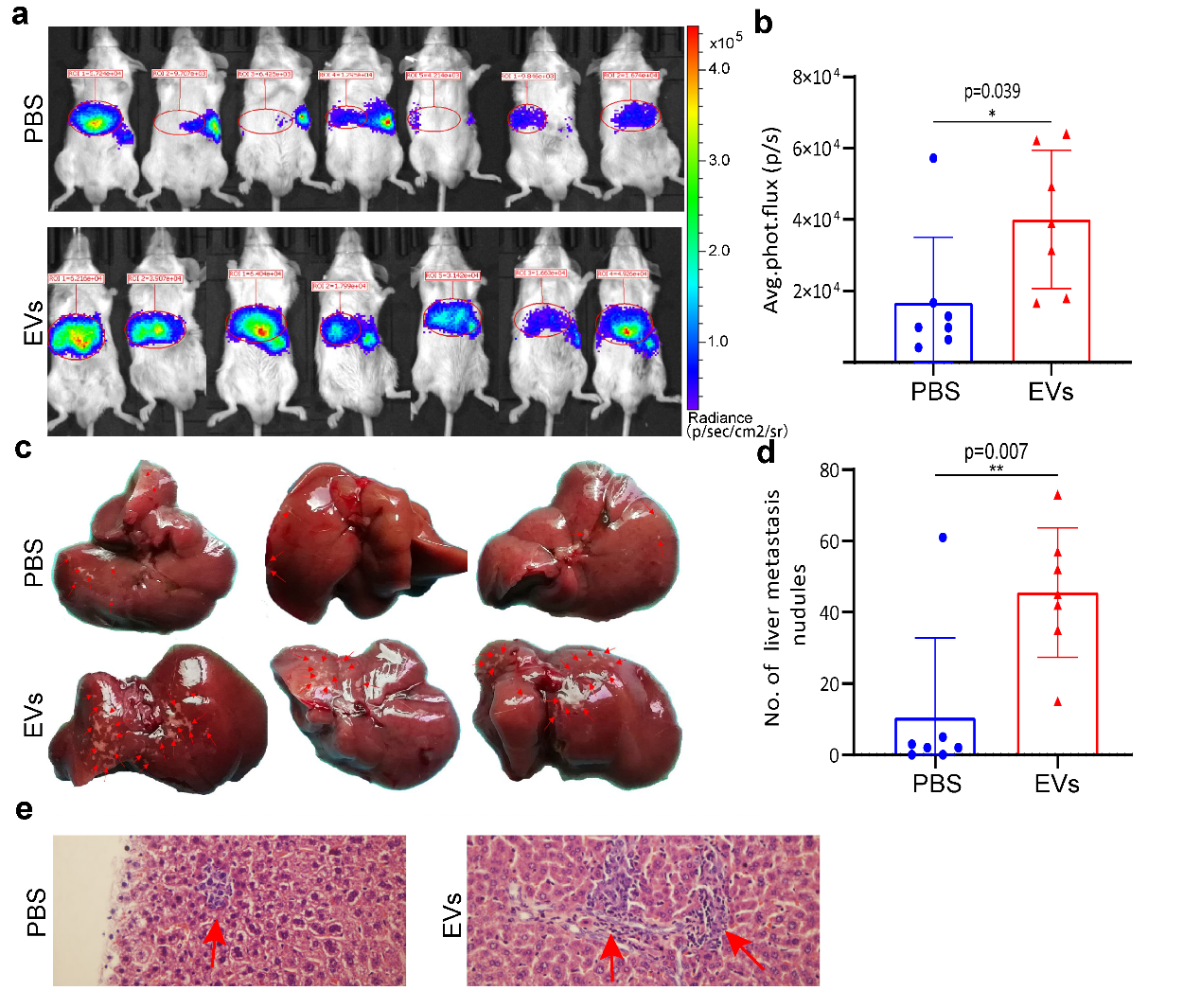


**Fig. S3: CRC-derived EVs promote tumor cells early liver metastasis under the condition of spleen preservation.** After mice were educated with CRC-derived EVs for 4 weeks, a CRC liver metastasis model was established with spleen preservation. a/b, The liver tumor fluorescence intensity was still significantly stronger in the EVs group at 24 h after operation; c/d, Representative images showed that the number of liver tumor nodules on the liver surface was significantly higher in the EVs group at 24 h after surgery. Statistical analysis confirmed that the tumor burden was heavier in the EVs group. e, The mice were sacrificed on day 4 after the operation. The liver tissue was fixed in formalin and embedded in paraffin. Liver tissue were stained with H&E. H&E staining confirmed that the white nodules were tumor nodules at 24 h after surgery. Data are shown as means ± standard deviations. * *p* < 0.05; ** *p* < 0.01, *** *p* < 0.001.

**Supplementary S4：**


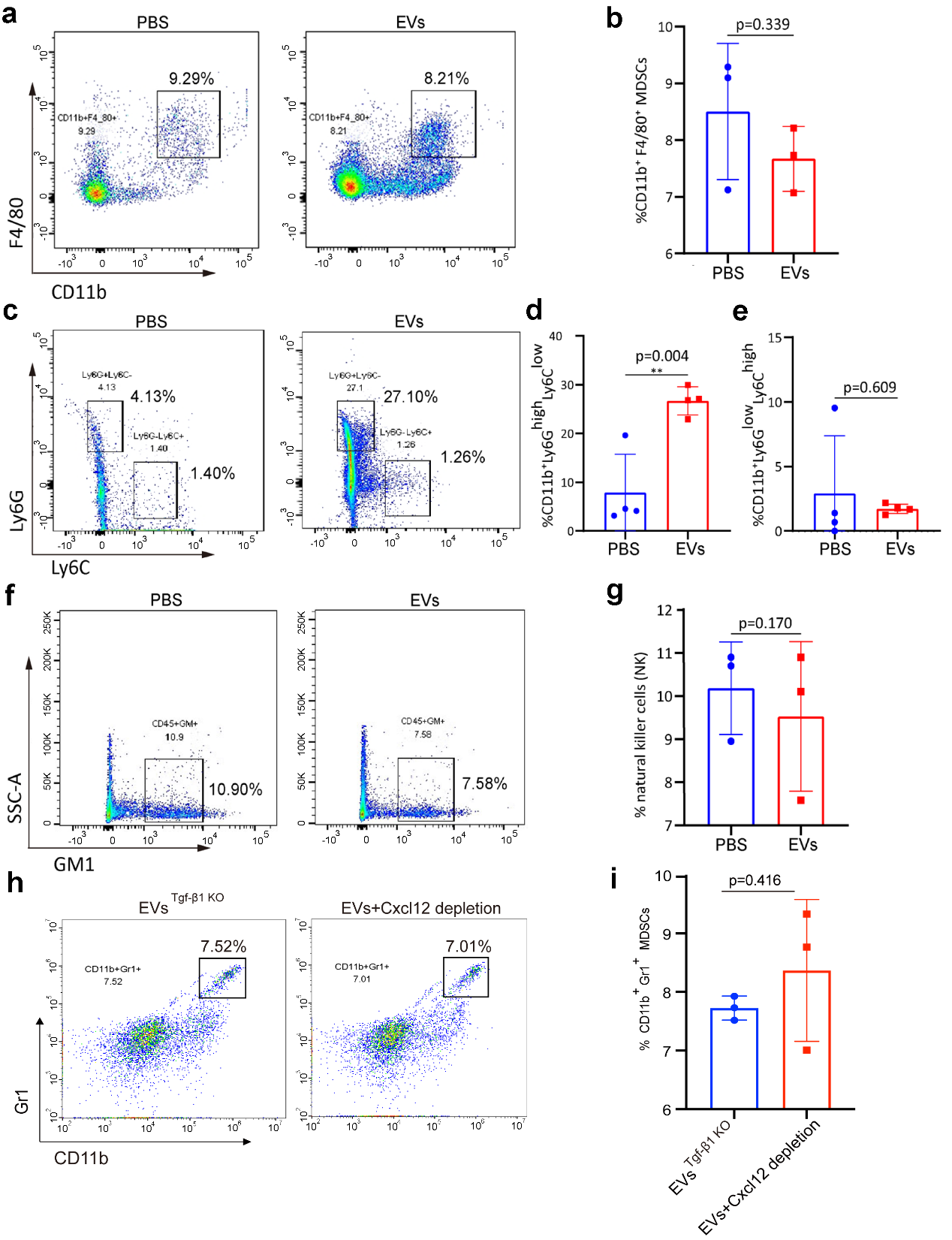


**Fig. S4: CRC-derived EVs remodel the liver premetastatic** **immunosuppressive niche.** a, Representative image demonstrated that CD11b^+^F4/80^+^ cells population screened by flow cytometry in the liver PMN did not change after CRC-derived EVs education (n = 3 in the PBS group, n = 3 in the EVs group). b, Quantitative analysis showed that no significant difference was found in the mean proportion of CD11b^+^ F4/80^+^ cells subset in the CD45^+^ lymphocyte population between the PBS group and the EVs group (8.50% vs 7.67%, *p* = 0.339). c, Representative image showed the CD11b^+^Ly6G^high^Ly6C^low^ cells subsets and CD11b^+^Ly6G^low^Ly6C^high^ cells subsets distribution in the liver PMN between the PBS group (n = 4) and the EVs group (n = 4). d/e, Statistical quantitative analysis showed that CD11b^+^Ly6G^high^Ly6C^low^ cells subsets, but not CD11b^+^Ly6G^low^Ly6C^high^ cells subsets, significantly increased after CRC-derived EVs education in the liver PMN. The mean proportion of CD11b^+^Ly6G^high^Ly6C^low^ cells subsets in the CD45^+^ lymphocyte population was 7.86% in the PBS group and 26.68% in the EVs group (*p* = 0.004). The mean proportion of CD11b^+^Ly6G^low^Ly6C^high^ cells subsets in the CD45^+^ lymphocyte population was 2.91% in the PBS group and 1.72% in the EVs group (*p* = 0.609). f, Representative image demonstrates that the CD45^+^GM1^+^ NK cells population in the liver PMN did not change after CRC-derived EVs education. g, Quantitative analysis showed that the mean proportion of CD45^+^GM1^+^ NK cells subset in the CD45^+^ lymphocyte population did not change significantly between the PBS group (n = 3) and the EVs group (n = 3) (10.18% vs 9.53%, *p* = 0.170). h, Representative image demonstrates that the CD11b^+^ Gr1^+^ cells subset in the liver PMN did not change after CRC-derived exosomal TGF-β1 knockout and HSCs-derived CXCL12 depletion. i, Quantitative analysis showed that the mean proportion of CD11b^+^ Gr1^+^ cells subset in the CD45^+^ lymphocyte population did not change significantly between the EVs TGF-β1 knockout group (n = 3) and the EVs with CXCL12 depletion group (n = 3) (7.92% vs 8.86%%, *p* = 0.416). Data are shown as means ± standard deviations. * *p* < 0.05; ** *p* < 0.01, *** *p* < 0.001.

**Supplementary S5：**


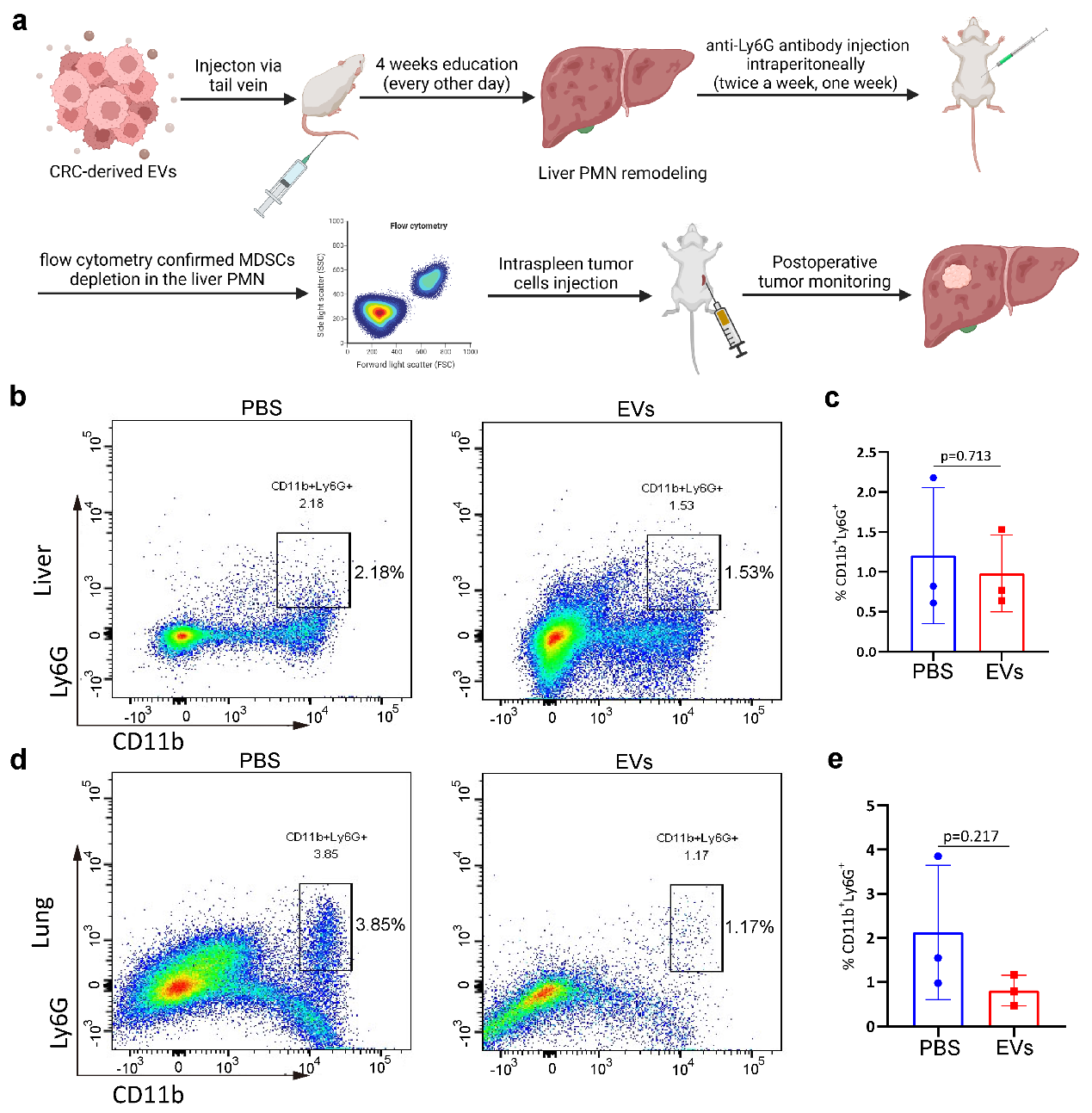


**Fig. S5: The role of recruited MDSCs.** a, Schematic study overview. b/d, Representative image showed the proportion of CD11b^+^Gr1^+^ cells population in the liver and lung PMN in the two groups after anti-Ly6G antibody injection. c/e, After PBS and EV education for one month and before anti-Ly6G antibody injection, the mean proportion of CD11b^+^Gr1^+^ cells population in the liver PMN was 7.09% in the PBS group and 14.17% in the EV group (*p* = 0.012, shown in Fig. 1d). Quantitative analysis showed that, after anti-Ly6G antibody injection, the mean proportion of CD11b^+^Gr1^+^ cells population in the liver and lung PMN decreased in both groups. In the liver PMN, the mean proportion of CD11b^+^Gr1^+^ cells population was 1.20% in the PBS group (n=3) and 0.98% in the EV group (n = 3) (*p* = 0.713). In the lung PMN, the mean proportion of CD11b^+^Gr1^+^ cells population was 2.13% in the PBS group (n = 3) and 0.81% in the EV group (n = 3) (*p* = 0.217). These results demonstrated no significant differences in the CD11b^+^Gr1^+^ cells population between the two groups after anti-Ly6G antibody injection. Data are shown as means ± standard deviations. **p* < 0.05; ** *p* < 0.01, *** *p* < 0.001.

**Supplementary S6 ：**


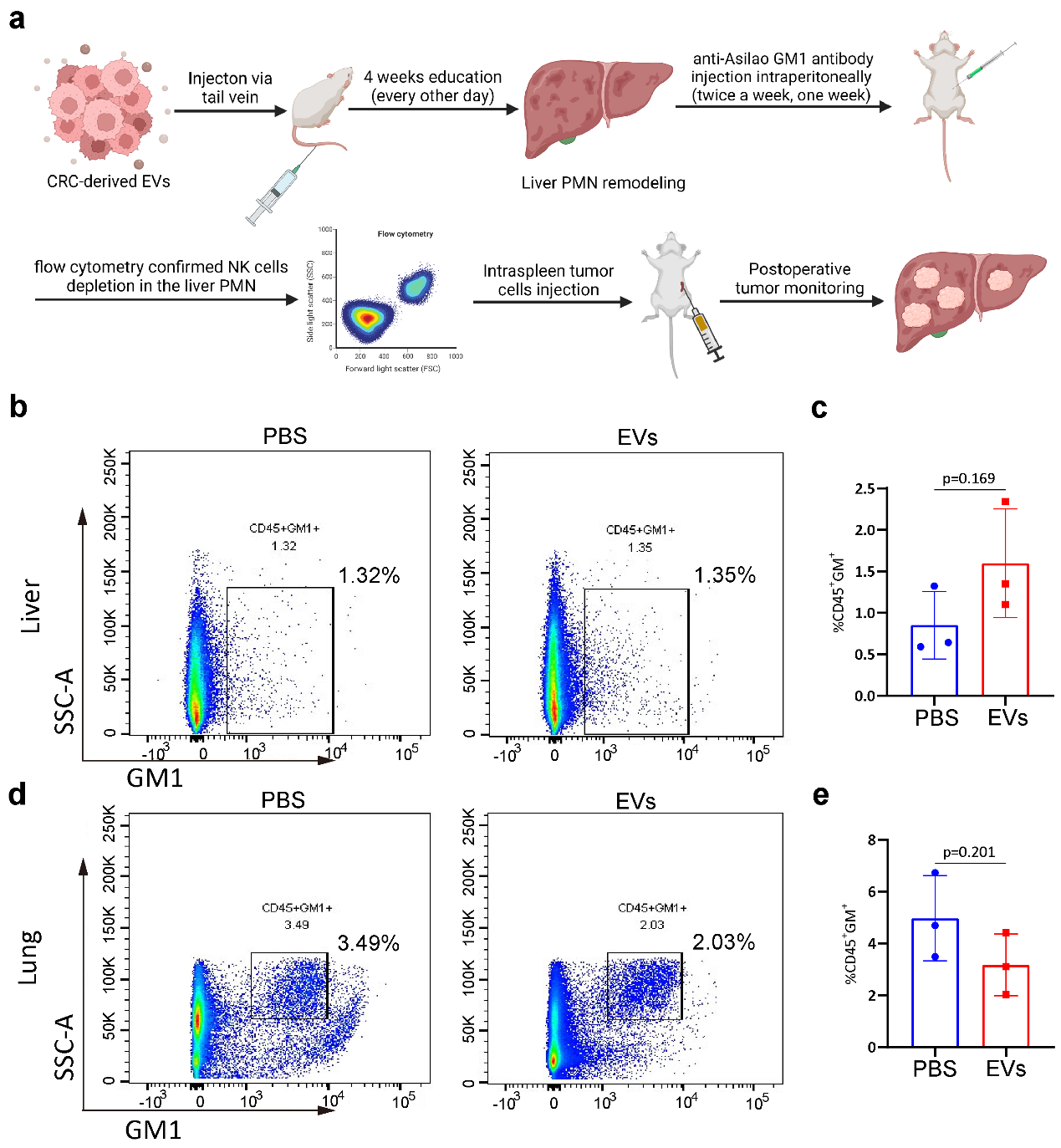


**Fig. S6: The role of NK cells in the liver PMN.** a, Schematic study overview. b/d, Representative image showed the proportion of CD45^+^GM1^+^ cells population in the liver and lung PMN after anti-asialo GM1 antibody injection. c/e, After PBS and EVs education for one month and before anti-asialo GM1 antibody injection, the mean proportion of CD45^+^GM1^+^ cells subset in the CD45^+^ lymphocyte population was 10.18% in the PBS group and 9.53% in the EVs group (*p* = 0.170, shown in Fig.S4f, g). Quantitative analysis showed that, after anti-asialo GM1 antibody injection, the mean proportion of CD45^+^GM1^+^ cells subset in the liver and lung PMN decreased in both groups. In the liver PMN, the mean proportion of CD45^+^GM1^+^ cells subset was 0.85% in the PBS group (n = 3) and 1.60% in the EVs group (n = 3) (*p* = 0.169). In the lung PMN, the mean proportion of CD45^+^GM1^+^ cells subset was 4.97% in the PBS group (n = 3) and 3.18% in the EVs group (n = 3) (*p* = 0.201). These results demonstrated no significant differences in the CD45^+^GM1^+^ cells subset between the two groups after anti-asialo GM1 antibody injection. Data are shown as means ± standard deviations. * *p* < 0.05; ** *p* < 0.01, *** *p* < 0.001.

**Supplementary S7：**


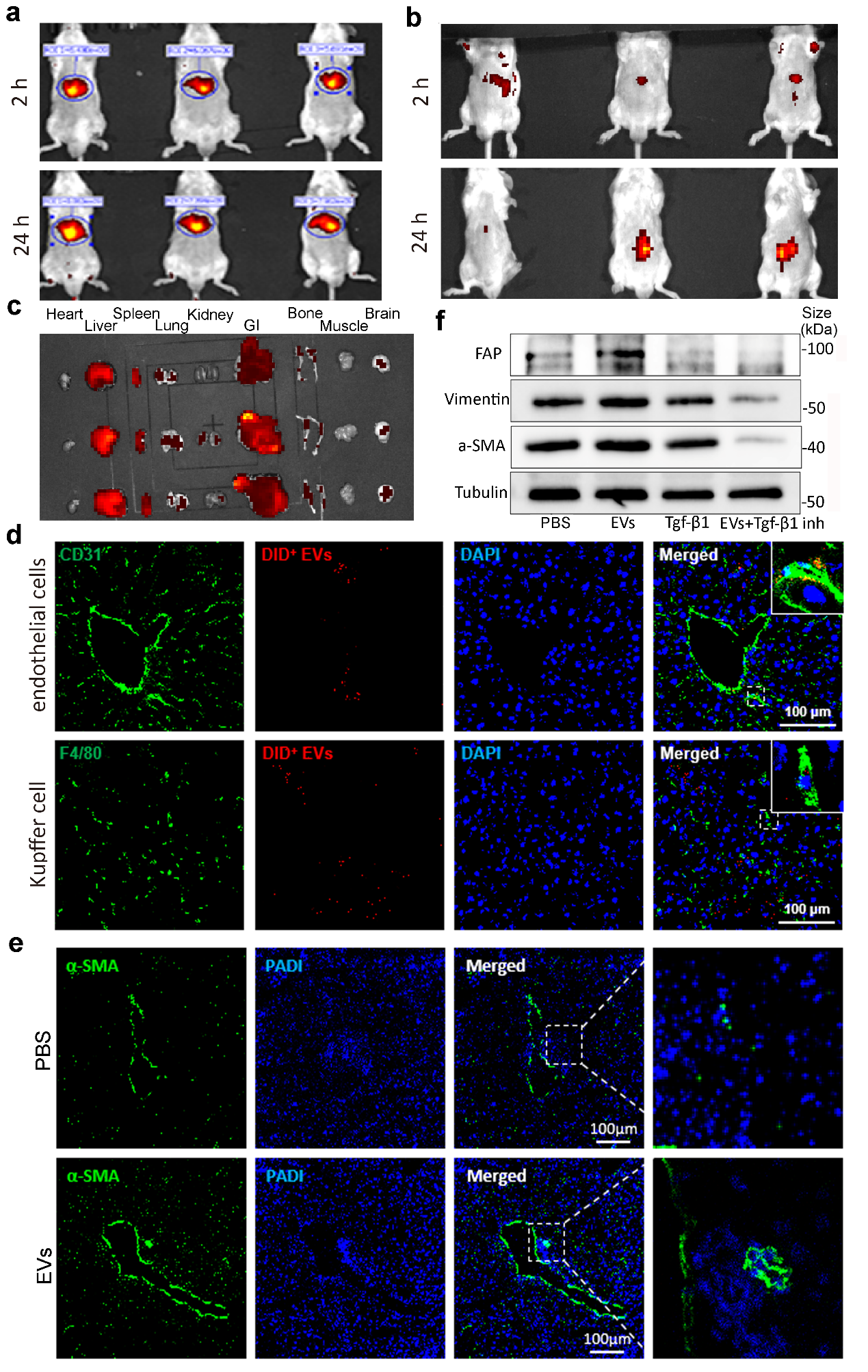


**Fig. S7: The investigation of EV distribution in vivo.** a, In vivo imaging analyses showed that DID dye–labeled CRC-derived EVs mainly accumulated in the liver at 2 and 24 h after injection. b/c, DiD^+^ PBS dye was used as a negative control, and the same volume (100 µL) of DiD-labeled PBS was injected into male BALB/c mice via tail vein. Two hours after injection, a small amount of DiD^+^ PBS dye accumulated in the liver, but mainly in the intestine after 24 h. It demonstrated that DiD^+^ PBS dye did not stay in the liver for a long time and was quickly excreted into the intestine. However, DiD^+^ EVs mainly accumulated in the liver and functioned persistently. d, Immunofluorescence confocal analysis demonstrated that DID dye–labeled EVs were mainly absorbed by Desmin^+^ HSCs cells. e, Immunofluorescence confocal analysis demonstrated that HSCs were activated and secreted little α-SMA in the Diss space after CRC-derived EVs education for 2 weeks. f, Western blotting further confirmed that CAFs markers (α-SMA, vimentin, and FAP) were induced by EVs and exogenous Tgf-β1. GI, gastrointestinal tract; Data are shown as means ± standard deviations. **p* < 0.05; ***p* < 0.01, ****p* < 0.001.

**Supplementary S8 ：**


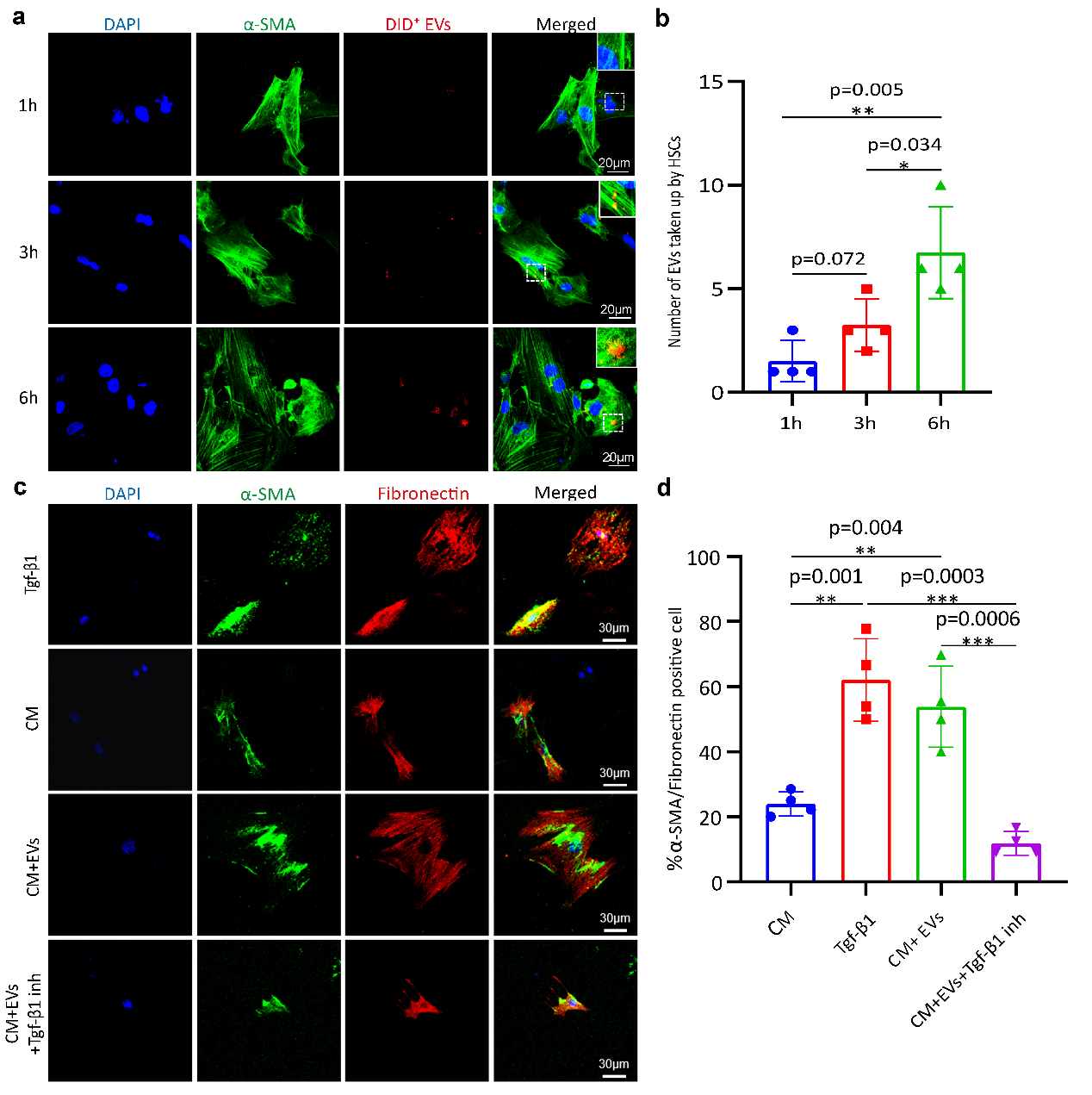


**Fig. S8: CRC-derived EVs carrying Tgf-β1 induce HSCs activation in vitro.** a/b, Immunofluorescence confocal analysis in vitro demonstrated that quiescent HSCs absorbed EVs, and the intake increased with the increase in culture time. c/d, Immunofluorescence confocal analysis demonstrated that, under in vitro co-culture condition, exogenous Tgf-β1 and CRC-derived EVs significantly activated HSCs and promoted fibronectin and α-SMA secretion. This means that HSCs were transformed into the CAFs phenotype, but the Tgf-β1 inhibitor abolished the effect. Inh, inhibitor; CM, culture medium; HSCs, hepatic stellate cells. Data are shown as means ± standard deviations. **p* < 0.05; ***p* < 0.01, ****p* < 0.001.

**Supplementary S9：**


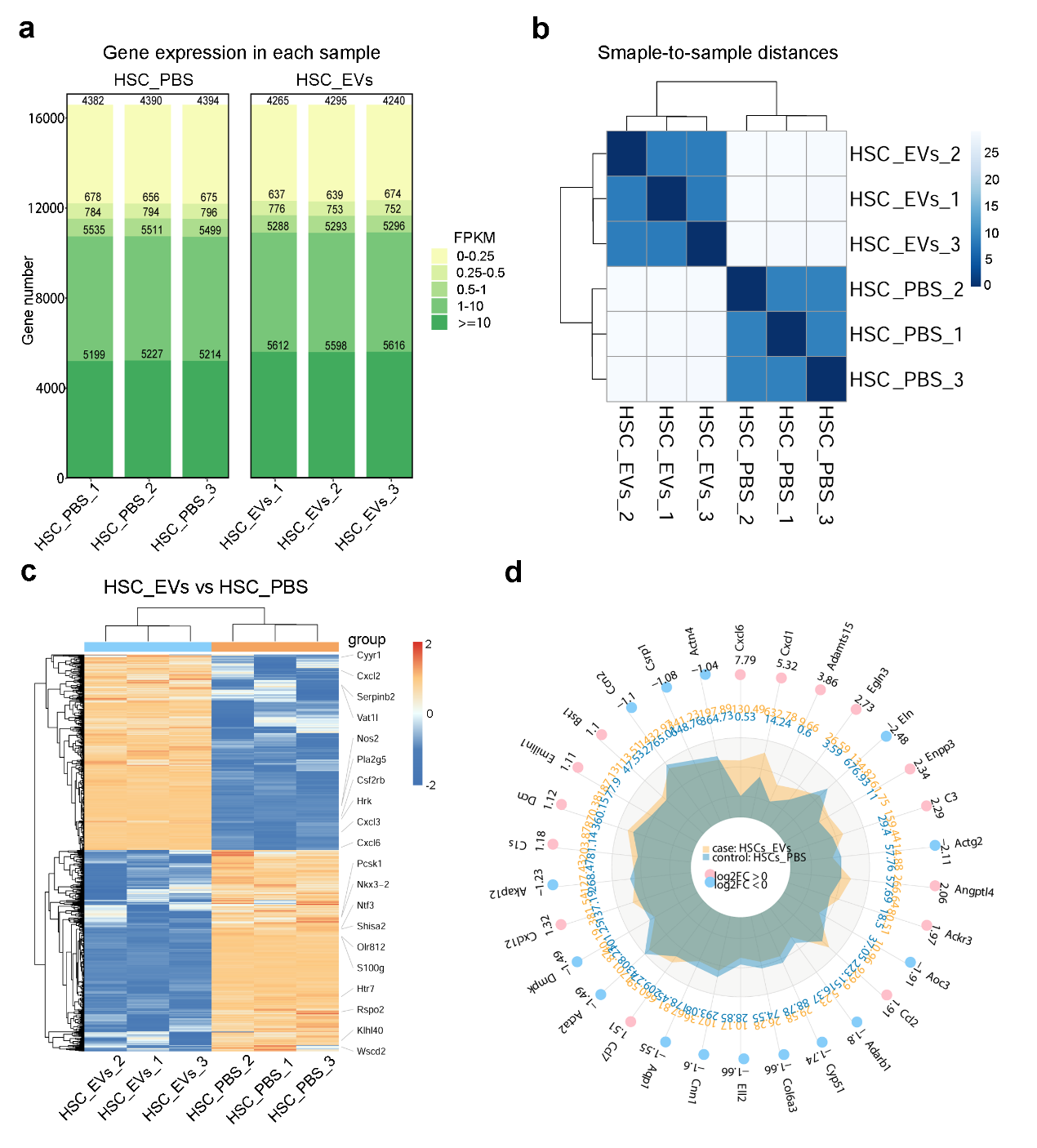


**Fig. S9: RNA sequencing reveals the mechanism of HSCs activation by CRC-derived EVs.** After HSCs were incubated with CRC-derived EVs for 48 h, RNA sequencing was performed to investigate the gene expression profiling. a, Gene expression in samples was shown, and for each sample we obtained over 10,000 transcripts. b, The correlation between samples was acceptable. c/d, There were 423 downregulated and 435 upregulated genes. The heat map and Radar map showed that chemokines, including CXCL 12, were highly expressed.

**Supplementary S10 ：**


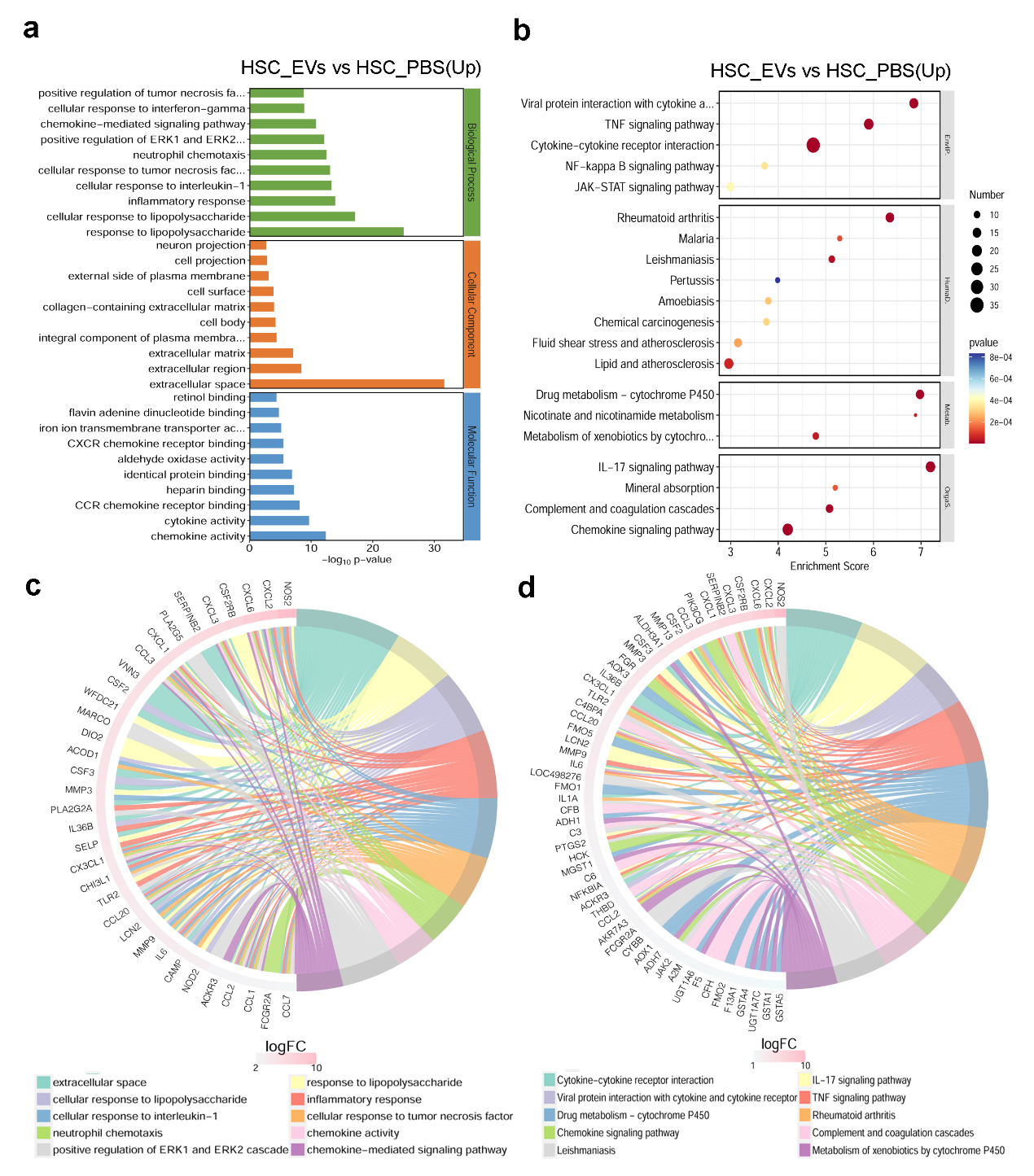


**Fig.S10**: **RNA sequencing reveals the mechanism of HSCs activation by CRC-derived EVs.** After HSCs were incubated with CRC-derived EVs for 48 h, RNA sequencing was performed to investigate the gene expression profiling. a/b, The GO analysis showed that the most upregulated genes involved chemokine activity and cytokine activity. c/d, The KEGG analysis showed that most upregulated genes were enriched in the chemokine signaling pathway.

**Supplementary S11 ：**


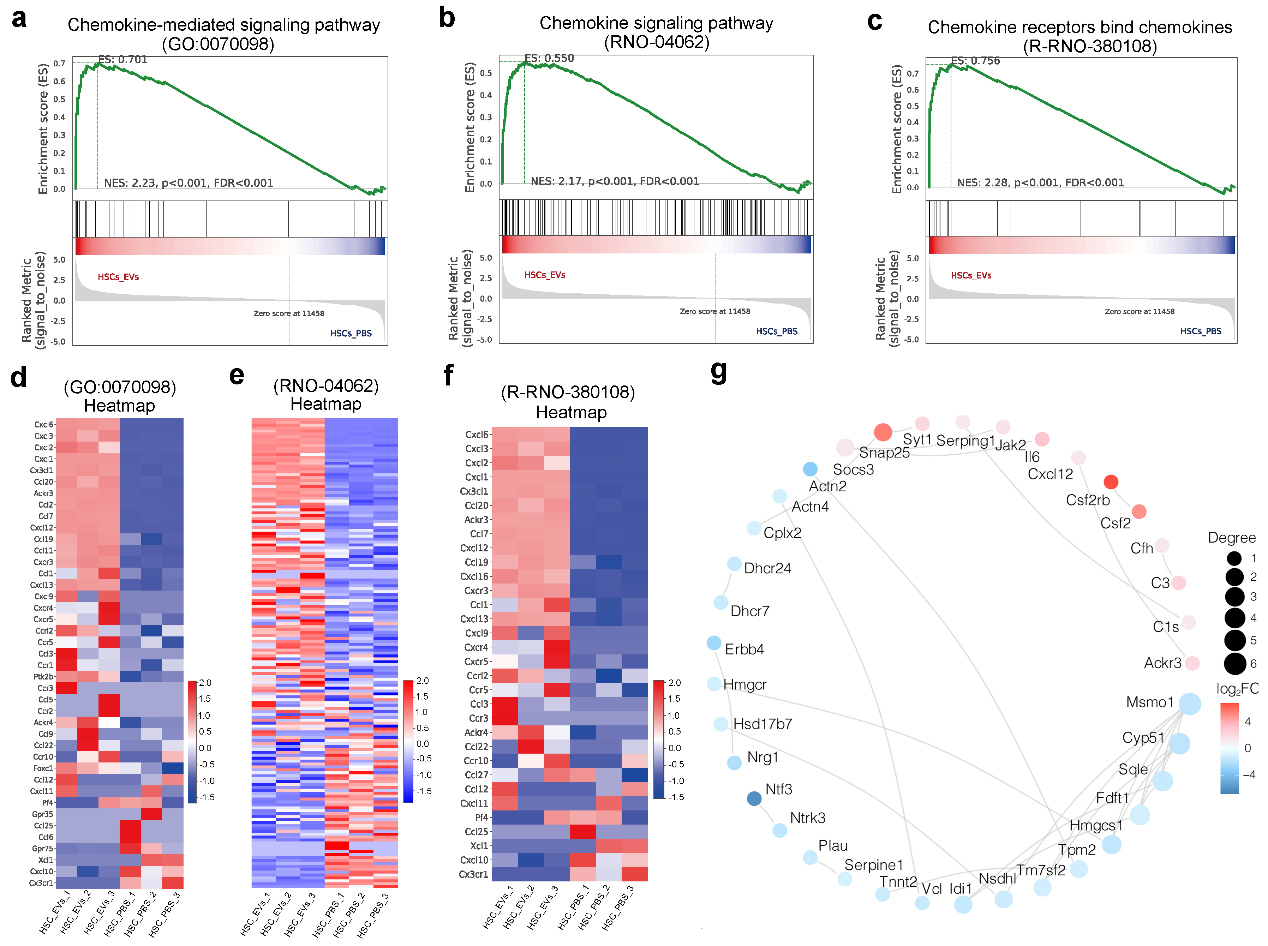


**Fig.S11: Gene Set Enrichment Analysis (GSEA) and Protein Interaction Network Analysis (PPI).** a–f, GSEA showed that the upregulated gene set was enriched in the chemokine-mediated signaling pathway and had the highest enrichment score. g, PPI showed that CXCL12 mainly interacted with ACKR3, also known as C-X-C chemokine receptor type 7 (CXCR-7).

**Supplementary S12：**


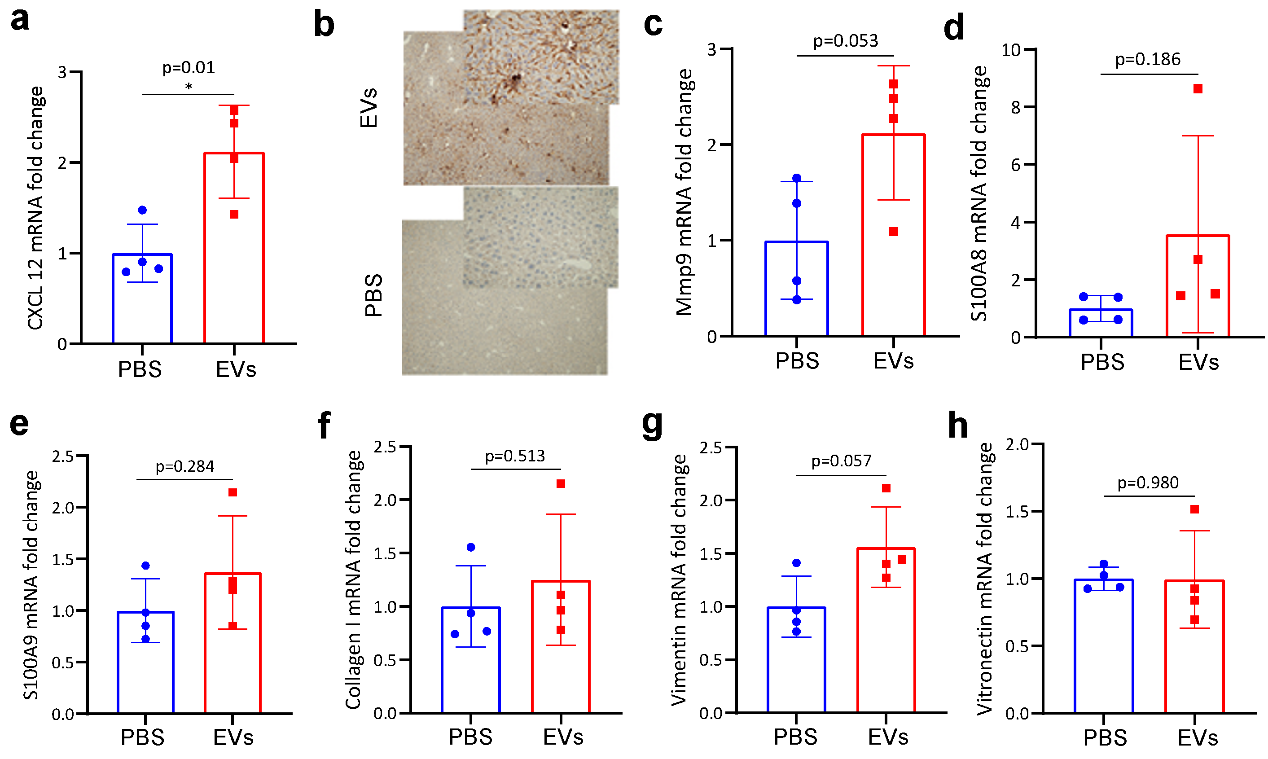


**Fig. S12: The expression of CXCL12 in activated HSCs.** a, qPCR showed that CXCL12 mRNA in the liver PMN was highly expressed. b, IHC staining further confirmed that CXCL12 was highly expressed in the liver PMN. c–h, qPCR was further used to investigate the expression levels of other inflammatory proteins in the liver PMN, including Mmp9, S100A8, S100A9, collagen I, vimentin, and vitronectin. The expression levels of MMP9 and vimentin tended to increase. Data are shown as means ± standard deviations. **p* < 0.05; ***p* < 0.01, ****p* < 0.001.

**Supplementary S13：**


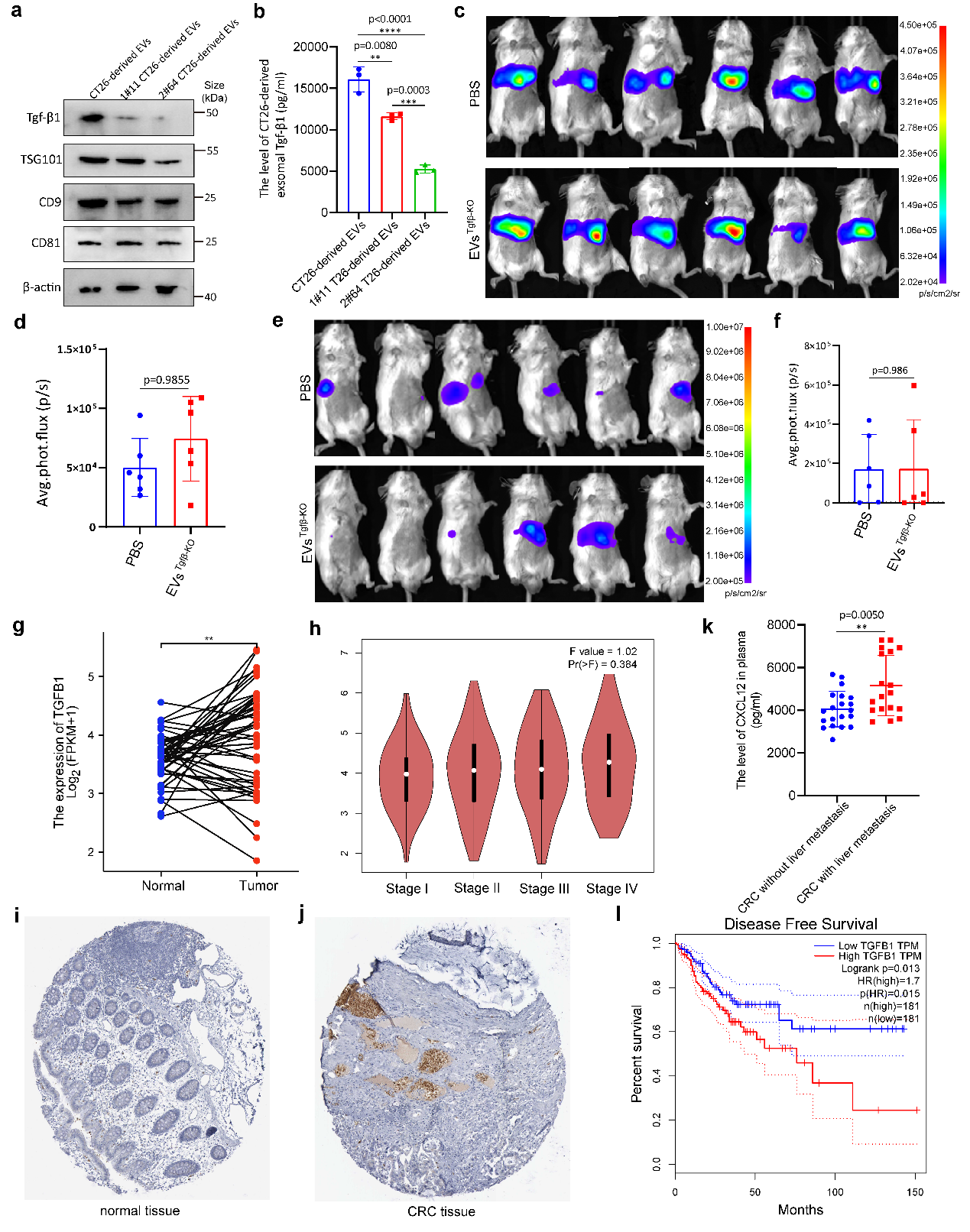


**Fig. S13: The effect of TGFB1 on tumor liver metastasis and CRC patients.** a, Western blotting confirmed that TGFB1 was successfully knocked out in CT26-derived EVs via CRISPR/Cas9 method. CT26-derived EVs without TGFB1 still expressed typical EVs markers, including CD9, CD81, and TSG101. b, ELISA further confirmed that the TGFB1 expression level in CT26-derived EVs was significantly downregulated. c/d, After mice were educated with Tgf-β1 knockout EVs for 4 weeks, a CRC liver metastasis model was established to confirm the effect of Tgf-β1 knockout. The liver metastasis model was successfully established in the PBS group (n = 6) and EVs Tgf-β1 group (n = 6), and liver tumor fluorescence intensity showed no significant difference between the two groups at 2 h after operation. e/f, There was no significant difference in tumor burden between the two groups at 24 h after operation. g, Bioinformatics analysis showed that the expression of TGFB1 was significantly increased in human CRC tissue. h, More advanced tumors tended to have higher TGFB1 expression levels than early tumors. i/j, IHC staining showed that human CRC tissue had a higher TGFB1 protein expression level. k, m/n, The expression levels of exosomal TGFB1 and CXCL12 in plasma were significantly higher in CRC patients with synchronous liver metastasis (n = 18) than in those without metastasis (n=20). l, Survival analysis suggested that patients with higher TGFB1 expression levels had worse disease-free survival. Data are shown as means ± SD. **p* < 0.05; ***p* < 0.01, ****p* < 0.001.

| **Supplementary table S1. The primers information.** | |
| --- | --- |
| primer name | Primer sequence |
| m CXCL12 RT F | GGAGGATAGATGTGCTCTGGAAC |
| m CXCL12 RT R | AGTGAGGATGGAGACCGTGGTG |
| m S100A8 RT F | CAAGGAAATCACCATGCCCTCTA |
| m S100A8 RT R | ACCATCGCAAGGAACTCCTCGA |
| m S100A9 RT F | TGGTGGAAGCACAGTTGGCAAC |
| m S100A9 RT R | CAGCATCATACACTCCTCAAAGC |
| m Mmp9 RT F | GCTGACTACGATAAGGACGGCA |
| m Mmp9 RT R | TAGTGGTGCAGGCAGAGTAGGA |
| m Collagen I RT F | CCTCAGGGTATTGCTGGACAAC |
| m Collagen I RT R | CAGAAGGACCTTGTTTGCCAGG |
| m vitronectin RT F | TGCTGCCTTCACTCGCATCAAC |
| m vitronectin RT R | GTCTGGTATGCCACTGAAGCCT |
| m Vimentin RT F | CGGAAAGTGGAATCCTTGCAGG |
| m Vimentin RT R | AGCAGTGAGGTCAGGCTTGGAA |
| Actin-F | GTCCCTCACCCTCCCAAAAG |
| Actin-R | GCTGCCTCAACACCTCAACCC |

**REFERENCES**

1. Moynihan KD, et al. Eradication of large established tumors in mice by combination immunotherapy that engages innate and adaptive immune responses. Nat Med. 22,1402-1410 (2016).
2. Nishikado H, et al. NK cell-depleting anti-asialo GM1 antibody exhibits a lethal off-target effect on basophils in vivo. *J Immunol*. 186,5766-5771 (2011).
3. Z. Tang, et al. GEPIA2: an enhanced web server for large-scale expression profiling and interactive analysis, Nucleic acids research. 47, W556-w560 (2019).

M. Uhlén, et al. Proteomics. Tissue-based map of the human proteome, Science (New York, N.Y.). 347, 1260419 (2015).
